# Identification of GATA Transcription Factors in *Brachypodium distachyon* and Functional Characterization of *BdGATA13* in Drought Tolerance and Response to Gibberellins

**DOI:** 10.3389/fpls.2021.763665

**Published:** 2021-10-21

**Authors:** Jie Guo, Xionghui Bai, Keli Dai, Xiangyang Yuan, Pingyi Guo, Meixue Zhou, Weiping Shi, Chenyang Hao

**Affiliations:** ^1^College of Agronomy, Shanxi Agricultural University, Jinzhong, China; ^2^Key Laboratory of Crop Gene Resources and Germplasm Enhancement, Ministry of Agriculture and Rural Affairs/The National Key Facility for Crop Gene Resources and Genetic Improvement/Institute of Crop Sciences, Chinese Academy of Agricultural Sciences, Beijing, China; ^3^Tasmanian Institute of Agriculture, University of Tasmania, Hobart, TAS, Australia

**Keywords:** *BdGATA13*, *Brachypodium distachyon*, drought stress, gene expression, transgenesis

## Abstract

GATA transcription factors (TFs) are type IV zinc-finger proteins that have roles in plant development and growth. The 27 GATA TFs identified in the *Brachypodium distachyon* genome in this study were unevenly distributed across all five chromosomes and classified into four subgroups. Phylogenesis-related GATAs shared similar gene structures and conserved motifs. Expression profiles showed that all *BdGATA* genes were expressed in leaves and most were induced by PEG treatment. *BdGATA13* was predominantly expressed in leaf tissue and phylogenetically close to *OsSNFL1*, *AtGNC*, and *AtGNL*. Its protein was detected in the nucleus by subcellular localization analysis. Overexpression of *BdGATA13* in transgenic *Arabidopsis* resulted in darker green leaves, later flowering, and more importantly, enhanced drought tolerance compared to the wild type. *BdGATA13* also promoted primary root development under GA treatment. These results lay a foundation for better understanding the function of *GATA* genes in *B. distachyon* and other plants.

## Introduction

GATA transcription factors (TFs), which have the consensus sequence W-G-A-T-A-R (*W* = T or A, *R* = G or A), are a class of regulators that exist in plants, fungi, and metazoans (Block and Shapira, 2015; Kobayashi and Masuda, 2016). The DNA-binding ligand of GATAs consists of a type IV zinc-finger motif with the consensus sequence CX_2_CX_17–20_CX_2_C followed by a basic region (Gupta et al., 2017). The first *GATA* gene *NTL1* containing a zinc finger motif with a C-X_2_-C-X_17_-C-X_2_-C sequence was identified in tobacco (*Nicotiana tabacum* L.) (Daniel-Vedele and Caboche, 1993).

Most studies have found that plant GATA TFs play an important role in plant development and growth. *GATA12* in *Arabidopsis* contributes to primary seed dormancy (Ravindran et al., 2017). An *Arabidopsis* B-GATA TF with an LLM domain in the C-terminus controlling leaf greening was characterized as a regulator of vegetative growth and development (Behringer et al., 2014; Bastakis et al., 2018); *GNC* regulates seed germination, stem elongation, and flowering time (Richter et al., 2010, 2013a; Chiang et al., 2012); and *HAN* is required to locate the proembryo boundary in early *Arabidopsis* embryos (Nawy et al., 2010). *HAN* is considered a boundary gene that regulates the development of shoot apical meristems and flower organs (Zhang et al., 2013). *Arabidopsis GNC* and *CGA1*, and rice (*Oryza sativa* L.) *Cga1* regulate chloroplast development (Chiang et al., 2012; Hudson et al., 2013). Overexpression of *OsGATA12* in rice causes increased leaf greenness, reduced leaf and tiller numbers, and ultimately affects yield-related traits (Lu et al., 2017). *OsGATA7* in rice modulates BR (brassinosteroid)-mediated regulation of plant architecture and grain shape (Zhang et al., 2018); *NL1* (*NECK LEAF 1*) regulates organogenesis during reproductive development in rice (Wang et al., 2009); and overexpression of *Cga1* caused semi-dwarf height and reduced tillering (Hudson et al., 2013). *Arabidopsis GATA12* is regulated by GA in a DELLA-dependent manner (Ravindran et al., 2017); *GNC* is implicated in regulation of carbon and nitrogen metabolism, and represses gibberellin signaling downstream of the DELLA proteins (Richter et al., 2010, 2013b; Chiang et al., 2012).

GATA TFs also respond to abiotic and biotic stresses. Expression profiles analysis show that rice, *Brassica juncea*, *Cucumis sativus*, and pepper *GATA* genes are in response to different abiotic stresses, including high temperature, salinity, cold, and drought treatments (Bhardwaj et al., 2015; Gupta et al., 2017; Yu et al., 2021; Zhang K. et al., 2021). *Arabidopsis GNC* and *GNL* participate in cold stress response (Richter et al., 2013a); overexpression of *OsGATA16* in rice improves cold tolerance (Zhang H. et al., 2021); overexpression of *OsGATA8* in rice improves drought tolerance (Nutan et al., 2020); and overexpression of *SlGATA17* improves drought tolerance in tomato (Zhao et al., 2021). Studies also show that the expression patterns of *AtGATA21*, *AtGATA22*, *OsGATA11*, *GmGATA44*, and *GmGATA58* are all inducible by nitrate (Scheible et al., 2004; Hudson et al., 2013; Zhang et al., 2015). And overexpression of wheat *TaGATA1* enhanced resistance to *Rhizoctonia cerealis* (Liu et al., 2020). The above reports indicate that a full assessment of GATA TFs in plants is needed due to their importance in development and growth as well as stress response.

Genome-wide analyses of GATA TFs in plant species identified 29 genes in *Arabidopsis*, 28 in rice, 35 in apple (*Malus* × *domestica* Borkh.), and 64 in soybean (*Glycine max* (L.) Merill.) (Reyes et al., 2004; Zhang et al., 2015; Chen et al., 2017). However, a systematic analysis of GATA TFs in the model grass *Brachpodium distachyon* has not occurred. In this study, 27 GATA TFs were identified in a genome-wide search in *B. distachyon* and the functions of *BdGATA13* in plant growth and in response to drought and GA treatments were investigated. The study sets a foundation for further studies on *GATA* genes.

## Materials and Methods

### Identification of *Brachpodium distachyon GATA* Genes

To identify GATA TFs genome-wide in *B. distachyon* we downloaded PF00320, a characteristic GATA domain, from the Pfam database (Mistry et al., 2021) and searched against the *B. distachyon* genome protein sequence. Twenty-nine *Arabidopsis* and 28 rice GATA protein sequences (Reyes et al., 2004) were used to BLAST (Basic Local Alignment Search Tool) against the genome protein sequence of *B. distachyon* with a threshold of <e^–5^ and identity of 50%. Redundant genes were manually removed before the NCBI-CDD and SMART programs were used to confirm that GATA TFs without a GATA domain were completely removed.

We used the ExPASy ProtParam^1^ to predict physicochemical properties of GATA TFs, and subcellular locations were predicted using CELLO v2.5^2^. The sequences of cDNAs, coding sequence (CDS), proteins, and DNA genes were extracted from the Ensembl Plants database^3^.

### Chromosome Location, Gene Duplication, and Phylogenetic Analyses

Gene location information was obtained from Ensembl Plants, and tandem and segmental duplication events were obtained from the PGDD (Plant Genome Duplication Database) (Lee et al., 2017) and visualized using TBtools (Chen et al., 2020). Un-rooted neighbour joining (NJ) and maximum likelihood (ML) trees were constructed using MEGA 7 with 1,000 bootstrap replications and the Poisson model based on the full-length protein sequences (Kumar et al., 2016), and visualized by Evolview v3 (Subramanian et al., 2019).

### Gene Structure and Conserved Motif Analyses

Gene structures of *BdGATA* genes were displayed by GSDS (Gene Structure Display Server) (Hu et al., 2015) after submitting the CDS and DNA sequences (Hu et al., 2015). MEME Suite (Bailey et al., 2009) was used to predict conserved motifs with the following parameters: number of motifs set at six, and width of motifs set from 6 to 50. The structures were visualized using Evolview v3 (Subramanian et al., 2019).

### Plant Growth and Stress Treatment, RNA Extraction, and cDNA Synthesis

Ten-day-old *B. distachyon* seedlings were planted in a growth chamber at 26/24°C (day/night) with a 14/10 h day/night photoperiod. Roots, stems, leaves, and spikes collected after heading were used for tissue expression analysis. To apply abiotic stresses 10-day-old seedlings were treated with salt (200 mM NaCl), drought (20% PEG), heat (45°C), cold (4°C), ABA (200 μM), and GA (10 μM) for 2 h in hydroponic culture to obtain whole plants for analysis. Materials were frozen in liquid nitrogen and stored at −80°C for further use. RNA extraction and cDNA synthesis were performed using the RNA Easy Fast Plant Tissue and FastKing gDNA Dispelling RT SuperMix (Tiangen Biotech, Beijing) Kits, respectively.

### Real-Time Quantitative-PCR

Real-time quantitative (qRT)-PCR was performed in triplicate using SuperReal PreMix Plus SYBR Green (Tiangen Biotech). Data collection and analyses were conducted using an ABI7900 system (Applied Biosystems, Germany). Data were normalized to *BdGAPDH* and *Atactin 8* as described previously (Hong et al., 2008; Reichel et al., 2016) and calculated using the 2^–ΔΔ*Ct*^ analysis method (Livak and Schmittgen, 2001). Primers used for PCR are listed in Supplementary Table 1.

### Vector Construction, Plant Transformation, and Subcellular Localization Assay

The full-length coding sequence of *BdGATA13* was amplified by PCR and cloned into the *pCambia*-1301 vector harboring the *CaMV35S* promoter. The recombinant vector was transformed into *Arabidopsis* strain *Col*-0 using the *GV3101*-mediated floral dip method (Clough and Bent, 1998). Transgenic lines were screened using a 0.1% hygromycin B solution and further confirmed by PCR. The full-length *BdGATA13* coding sequence without a stop codon was inserted into the *pCambia-1301-GFP* vector to produce construct 35S: *BdGATA13*-*GFP*. For subcellular localization assays this construct and the vector *pCAMBIA1301-GFP* were co-transformed into tobacco leaves. Subcellular localization in tobacco leaves using GFP and DAPI staining was expedited by confocal microscopy (Olympus IX83-FV1200, Japan).

### Tolerance Assays Under Stress Conditions

Seeds of wild type *Arabidopsis* and transgenic lines were surface-sterilized and sown on 1/2 MS plates and incubated in darkness at 4°C for 48 h before germination. Chlorophyll content was measured according to a previous study (Zhang et al., 2011). For phenotypic assessment under drought stress, 5-day-old seedlings were transplanted to 1/2 MS plates containing 0, 100 and 200 mM mannitol and cultured at 23°C in a 16/8 h (light/darkness) photoperiod for 10 days. Five-day-old seedlings for GA treatment were transplanted to 1/2 MS plates containing 0, 0.25 and 0.5 μmol GA_3_ and cultured under the above conditions for 5 days. There were six replicates of each treatment; root lengths for each sample were measured by ImageJ (Rueden et al., 2017); and data were analyzed using Microsoft Excel 2010. Error bars in all graphs represent means ± S.D and “^∗^” (*P* < 0.05) or “^∗∗^” (*P* < 0.01) were used to indicate significant differences found by Student’s *t*-tests.

## Results

### Identification of *Brachypodium distachyon* GATAs

**FIGURE 1:**
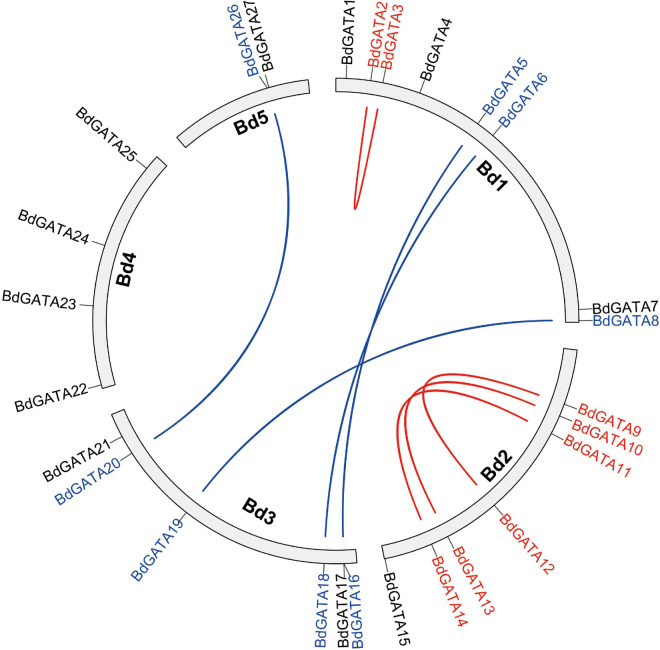
Chromosome locati on and analysis of duplicated *BdGATAs*. Red lines connect tandemly duplicated genes; blue lines connect segmentally duplicated genes.

Twenty-seven GATAs were identified in the *B. distachyon* genome. To facilitate subsequent analysis, these *GATA* genes were named *BdGATA1* to *BdGATA27* according to their chromosomal location. The *BdGATA* loci were unevenly distributed and there were 2 to 8 genes on each chromosome (Figure 1). Eight *BdGATA* genes were present as four tandem duplications; and eight were in four segmental duplications.

BLAST of the CDS of each gene against the *B. distachyon* EST database in NCBI^4^ revealed that seven *BdGATAs* had no EST validation. Characteristic features of each gene were further examined. Average molecular weights, isoelectric points, and grand average hydropathicities were 35.478 KDa, 7.68, and −0.63, respectively. Detailed information for each gene is provided in Supplementary Table 2.

### Phylogenetic Analysis of GATAs

**FIGURE 2:**
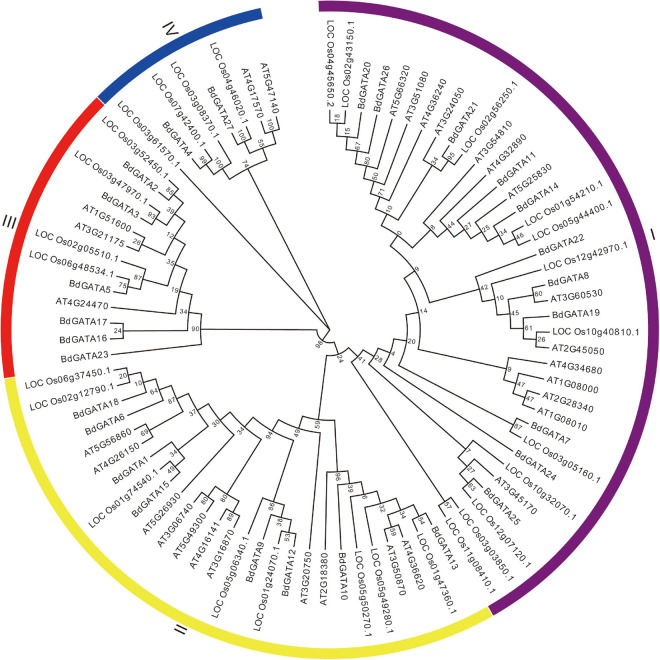
NJ tree of GATAs in plants. The tree included 27 GATAs from *Brachypodium*, 29 from *Arabidopsis* and 28 from rice, and construction was based on the full-length protein sequences. Four subgroups of GATAs were classified as I, II, III, and IV.

**FIGURE 3:**
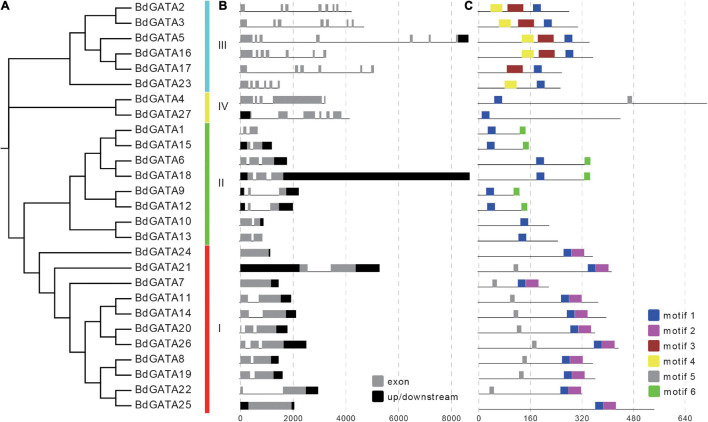
Phylogenetic, gene structure, and conserved motif analysis of *BdGATAs*. **(A)** The phylogenetic tree divided GATAs into four subgroups. **(B)** Structures of *BdGATA* genes. Black rectangles indicate up/down stream sequences; gray rectangles indicate exons; gray lines indicate introns. **(C)** Conserved motif analysis of BdGATAs. Different motifs are identified and displayed in different colors.

To elucidate the phylogenetic relationships among plant GATAs the 27 full-length GATA protein sequences in *B. distachyon*, 29 in *Arabidopsis*, and 28 in rice were extracted to build an un-rooted NJ tree. As shown in Figure 2 they were divided into four subgroups designated I, II, III, and IV based on bootstrap support. To further validate the reliability of the NJ tree the ML tree was also generated and formed the same subgroups (Supplementary Figure 1). The phylogenetically related genes were functionally conserved. For example, subgroup II members *AT5G56860* (*GNC*), *AT4G26150* (*GNL*), and *LOC_Os06g37450* (*OsGATA16*) were found to participate in response to cold stress (Richter et al., 2013a; Zhang H. et al., 2021); and *LOC_Os05g50270* (*SNFL1*) and *LOC_Os02g12790* (*Cga1*) regulated plant architecture (Hudson et al., 2013; He et al., 2018); Overexpression of subgroup II members *AT3G06740*, *AT3G16870*, *AT5G56860* (*GNC*), *AT4G26150* (*GNL*), *BdGATA15*, and *BdGATA18* in *Arabidopsis* showed increased chlorophyll accumulation and delayed flowering (Behringer et al., 2014). An unrooted NJ phylogenetic tree for *B. distachyon* placed 11, 8, 6, and 2 *BdGATAs* into subgroups I, II, III, and IV, respectively (Figure 3A).

### Gene Structure and Conserved Motif Analysis

Analysis of the gene structures and conserved motifs in BdGATAs (Figure 3B) showed that the numbers of exons in *BdGATA* genes ranged from one to eight; exon numbers in subgroups I and II varied from three to eight whereas subgroups III and IV had one to three. Six types of motifs were identified in BdGATA proteins (Figure 3C); motif one was present in all BdGATAs whereas motifs two, three, and six were specific to subgroups IV, I, and III, respectively. Motif one formed the GATA domain.

### Expression Pattern of *GATA* Genes

**FIGURE 4:**
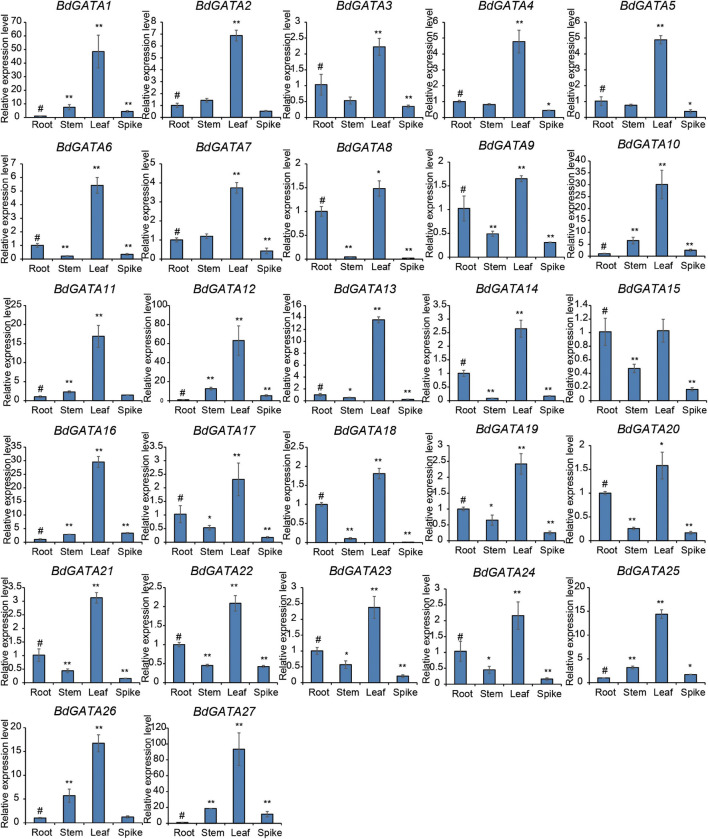
Expression patterns of *BdGATAs* in different tissues. The ordinate indicates the relative expression level, and the abscissa represents different tissues. The results were normalized against the expression of *BdGAPDH* as an internal control. Values are means ± SD (*n* = 3). Asterisks represent statistically significant differences between the indicated samples. Root (#) is used as control for each tissue. Student’s *t*-test: *P ≤ 0.05 and **P ≤ 0.01.

Analysis of the expression patterns of the *GATA* genes in root, stem, leaf, and spike tissues at different stages of plant development (Figure 4) showed that all *BdGATA* genes except *BdGATA15* had much higher expression levels in the leaves than in other tissues. There were much lower differences among roots, stems, and spikes with some genes showing higher expression levels in roots but others with higher expression levels in stems or spikes.

**FIGURE 5:**
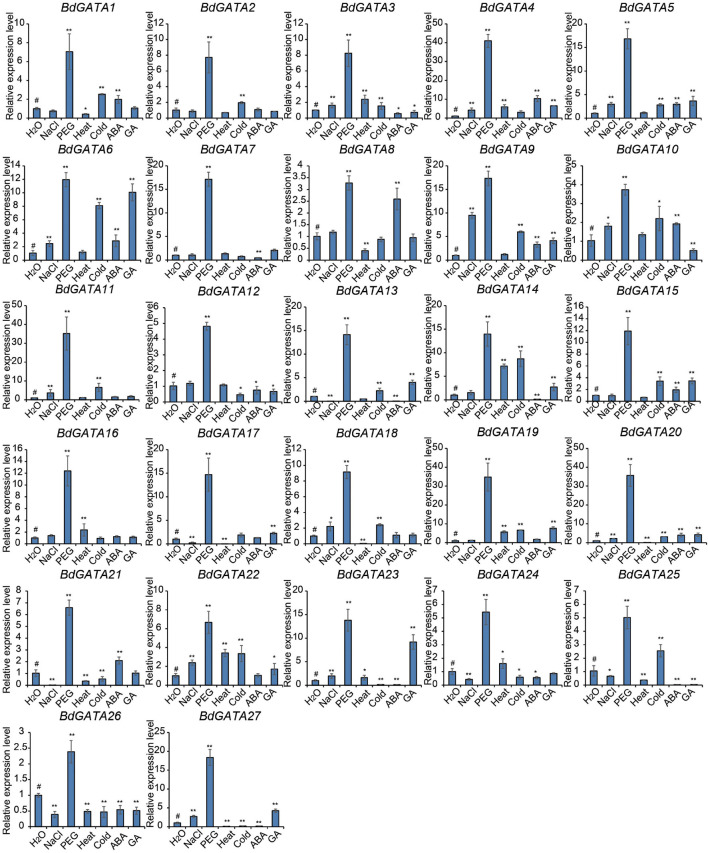
Expression patterns of *BdGATAs* under abiotic stresses. The ordinate indicates the relative expression level; and the horizontal axis represents different abiotic stresses, including salt (200 mM NaCl), drought (20% PEG), heat (45°C), cold (4°C), ABA (200 μM), and GA (10 μM) for 2 h in hydroponic culture. The results were normalized against the expression of *BdGAPDH* as an internal control. Values are means ± SD (*n* = 3). Asterisks represent statistically significant differences between the indicated samples. H_2_O (#) is used as control for each treatment. Student’s *t*-test: *P ≤ 0.05 and **P ≤ 0.01.

Gene expression under different abiotic stresses was also evaluated using RT-PCR (Figure 5). *BdGATA* genes also participated in abiotic stress responses. PEG treatment showed the greatest impact on expression of all the *BdGATA* genes. Other treatments caused changes in expression levels of some genes. For example, the expression levels of *BdGATA6*, *BdGATA9*, *BdGATA13*, *BdGATA14*, *BdGATA15*, *BdGATA19*, *BdGATA23*, and *BdGATA27* were significantly upregulated by GA treatment; *BdGATA6* and *BdGATA15* were significantly upregulated and downregulated by cold treatment, respectively; and genes *12* and *6* were significantly upregulated and downregulated by salt treatment, respectively. Some genes, such as *BdGATA26*, showed consistent down-regulation under most treatments while others, such as *BdGATA5* and *BdGATA9*, showed consistent up-regulation under most treatments.

### *BdGATA13* Is Located in the Nucleus

**FIGURE 6:**
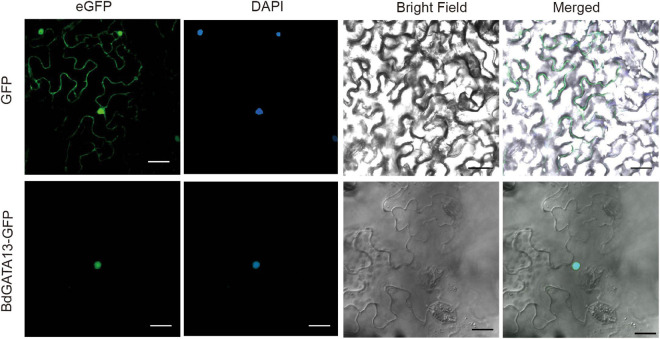
Subcellular localization of BdGATA13 protein. 35S: *BdGATA13*-GFP was transferred into tobacco leaves, and fluorescence signals of GFP were detected in tobacco leaf epidermal cells. Left panel, GFP image; middle panel, bright field; and right panel, merge of GFP and bright field. Bar, 10 μm.

A *BdGATA13*-eGFP fusion driven by the 35S promoter was transformed into tobacco leaves to investigate subcellular localization. The 35S:*BdGATA13*-eGFP fusion protein was detected in the nucleus (Figure 6), consistent with its predicted function as a TF.

### Overexpression of *BdGATA13* in *Arabidopsis* Increases Chlorophyll Content and Delays Flowering Time

**FIGURE 7:**
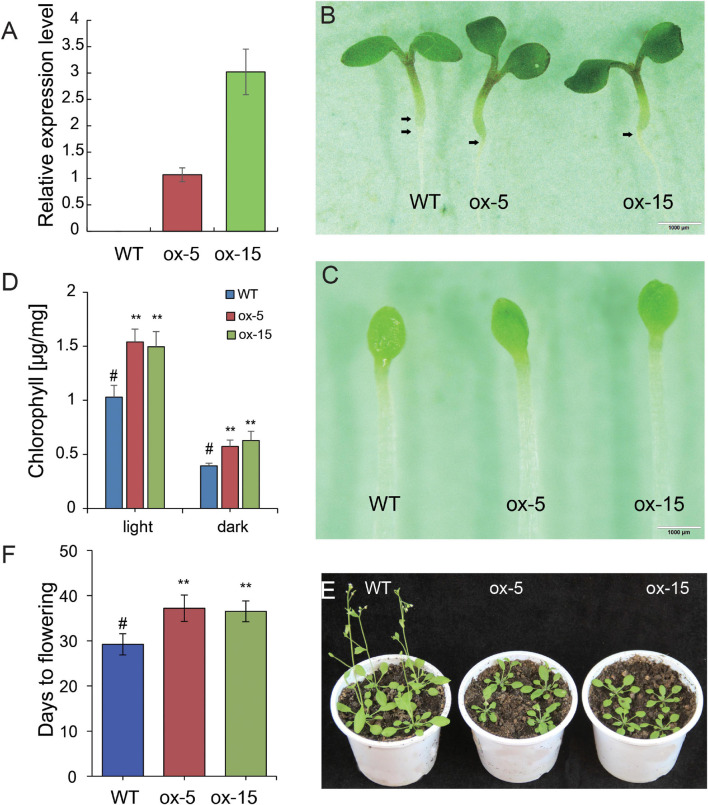
Effects of the ectopic expression of *BdGATA13* on greening and flowering in transgenic *Arabidopsis*. **(A)** Relative expression levels of two transgenic lines by qRT-PCR, N.D., not detected. **(B)** Phenotype of the WT and the two transgenic lines in 5-day-old seedlings grown under long-day conditions, bar = 1 mm. **(C)** Phenotypes of 5-day-old WT and two transgenic lines grown in darkness, bar = 1 mm. **(D)** Average and standard deviations of chlorophyll levels of WT and transgenic plants grown for 5 days in light or darkness (three biological replications). **(E)** Flowering time comparison of WT and transgenic lines grown in long-day conditions. **(F)** Comparison of days to flowering for WT and transgenic plants. Values are means ± SD (*n* = 3). Asterisks represent statistically significant differences between the indicated samples. Student’s *t*-test: *P ≤ 0.05, **P ≤ 0.01.

All *BdGATA* genes were highly expressed in leaves (Figure 4), indicating that *BdGATA* genes have an important role in leaf growth and development. As one example, we investigated the function of *BdGATA13*, a gene predominantly expressed in leaf tissue but not previously studied. This gene is phylogenetically close to subgroup II genes *LOC_Os05g50270* (*SNFL1*), *GNC* (*AT5G56860*), and *GNL* (*AT4G26150*). To further analyze whether their functions were conserved in the phylogeny, the CDS of *BdGATA13* driven by the 35S promoter was transformed into *Arabidopsis*, and two transgenic lines (ox-5 and ox-15) showing different gene expression levels (Figure 7A) were generated and used for phenotypic analyses. Overexpression of *BdGATA13* produced dark green seedling leaves (Figures 7B,C) by accumulation of chlorophyll (Figure 7D) under both dark and light conditions. Flowering time of the transgenic lines was also delayed (Figures 7E,F).

### *BdGATA13* Enhances Drought Tolerance in Transgenic *Arabidopsis*

**FIGURE 8:**
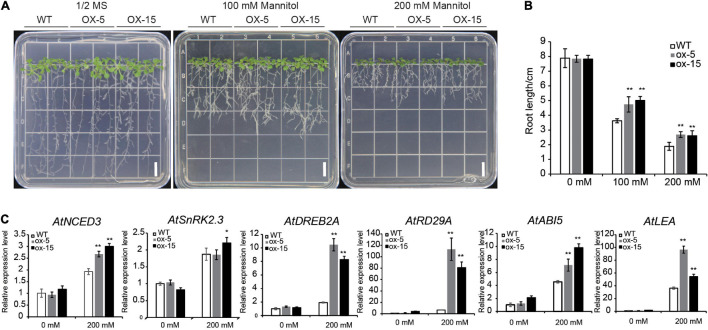
Overexpression of *BdGATA13* enhanced tolerance to drought stress in *Arabidopsis* seedlings. **(A)** 5-day-old of seedlings of *Arabidopsis* were grown in 0, 100, and 200 mM mannitol for 10 day; bars = 1 cm. **(B)** Primary root lengths of WT and transgenic lines. **(C)** qRT-PCR results for six drought-related genes under 0 and 200 mM mannitol. Values are means ± SD (*n* = 3). Asterisks represent statistically significant differences between the indicated samples. Student’s *t*-tests: *P ≤ 0.05, **P ≤ 0.01.

The expression level of *BdGATA13* was increased by PEG and GA treatments (Figure 5). Root growth of the transgenic lines on 1/2 MS medium was similar to wild type plants but was clearly increased relative to the WT under drought treatment (*P* < 0.01) (Figure 8A). When grown in 100 mM mannitol solution, the root lengths of the transgenic lines were increased by 30.70 and 38.08% compared to wild type plants (*P* < 0.01) (Figure 8B). When grown in 200 mM mannitol, the root lengths of the transgenic lines were increased by 42.41 and 38.19%, respectively (*P* < 0.01) (Figure 8B). These results clearly demonstrated that *BdGATA13* enhanced drought tolerance in *Arabidopsis*.

The expression levels of several drought-responsive genes, including *RD29A*, *ABI5*, *LEA*, *NCED3*, *SnRK2*.3, and *DREB2A*, were also determined. Except for *SnRK2.3*, expression levels of these genes were induced by drought treatment (Figure 8C). These results indicated potential links between *BdGATA13* and drought-related genes in *Arabidopsis*.

### *BdGATA13* Affects Plant Growth by Negative Regulation of GA Signaling

**FIGURE 9:**
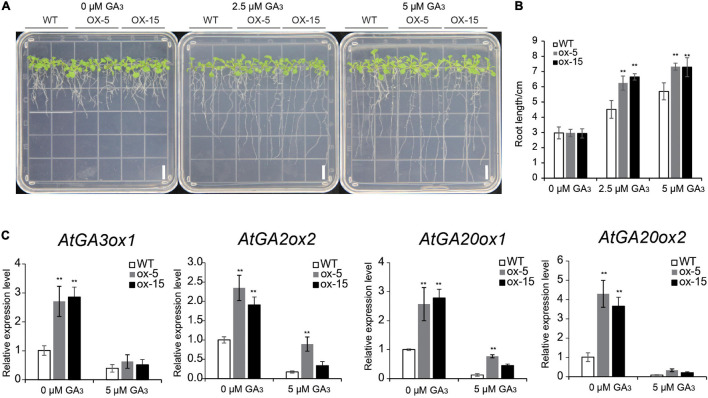
Overexpression of *BdGATA13* increased root length in *Arabidopsis* seedlings under GA_3_ treatment. **(A)** 5-day-old *Arabidopsis* seedlings were subjected to 0, 0.25, and 0.5 μmol GA_3_ treatment for 5 day; bars = 1 cm. **(B)** Comparison of primary roots in WT and transgenic lines. **(C)** qRT-PCR results for four gibberellin-related genes under 0 and 0.5 μmol GA_3_. Values are means ± SD (*n* = 3). Asterisks represent statistically significant differences between the indicated samples. Student’s *t*-tests: *P ≤ 0.05, **P ≤ 0.01.

The expression level of *BdGATA13* was upregulated by GA_3_ treatment. As shown in Figure 9A, the root lengths of the two transgenic lines did not differ from that of wild type plants under control conditions. However, under 2.5 μM GA_3_ treatment, the root lengths of the transgenic lines were 38.32–47.26% higher than the wild type (*P* < 0.01) and under 5 μM GA_3_ treatment, the root lengths of transgenic lines were 28.69% higher than the wild type (*P* < 0.01) (Figure 9B).

The expression levels of gibberellin-related genes *GA3ox1*, *GA2ox*, *GA20ox1*, and *GA20ox2* were also assayed. Expression levels of all four genes in the transgenic lines were higher than those in the WT without GA_3_ treatment (*P* < 0.01) (Figure 9C) and under 5 μM GA_3_ treatment expression levels of the four genes in transgenic plants were similar to the wild type (Figure 9C). These results suggested that *BdGATA13* affects plant growth by negative regulation of GA signaling.

## Discussion

### Characteristics of GATA in *Brachypodium distachyon*

We identified 27 GATA TFs in the *B. distachyon* genome, a similar number to *Arabidopsis* (29) (Reyes et al., 2004), rice (28) (Gupta et al., 2017), pepper (*Capsicum tetragonum* L.) (28) (Yu et al., 2021), but less than in *Brassica napus* (96) (Zhu et al., 2020), apple (*Melus pumila* L.) (35) (Chen et al., 2017), and soybean [*Glycine max* (L.) Merill] (64) (Zhang et al., 2015). Among the 27 *BdGATA* genes, 16 were duplicated, including eight tandemly duplicated and eight segmently duplicated. The expression levels of the segmently duplicated genes were similar in all tissues. For example, *BdGATA8* and *BdGATA19* were segmently duplicated and showed low expression in stem and splike tissues but high expression in leaf tissue. These results indicated that gene duplication contributed to expansion of the *GATA* gene family in *B. distachyon*. This corresponds with findings in apple (Chen et al., 2017), *Gossypium hirsutum* L. (Zhang et al., 2019), *B. napus* (Zhu et al., 2020), and pepper (Yu et al., 2021).

Phylogenetic analysis of rice, *Arabidopsis*, and *B. distachyon* GATAs identified four subgroups (Figure 2). In the case of *B. distachyon* there were 11, 8, 6, and 2 members in each subgroup and subgroups shared similar gene structures and conserved motifs, and implying conserved functions among members within each subgroup (Figure 3). Among conserved motifs, motif one was present in all BdGATA members and formed a GATA domain. Expression pattern analysis showed that all *BdGATA* genes were highly expressed in leaf tissues. Members in the same subgroup had similar functions. For example, subgroup II members *GNC* (*AT5G56860*) and *GNL* (*AT4G26150*) contribute to chlorophyll biosynthesis as evidensed by chlorophyll accumulation in grown *A. thaliana* seedlings grown in light (Bastakis et al., 2018). Overexpression of *Arabidopsis* subgroup II members *AT3G06740*, *AT3G16870*, *AT5G56860* (GNC), *AT4G26150* (GNL), *BdGATA15*, and *BdGATA18* showed increased chlorophyll accumulation and delayed flowering (Behringer et al., 2014). Subgroup II member *BdGATA13* also accumulated chlorophyll when grown in light, confirming that *BdGATA* genes in the same subgroups have similar functions.

### *BdGATA13* Regulates Plant Development and Responds to Stress

All *BdGATA* genes had relatively high expression levels in leaf tissues indicating a significant role of *GATA* genes in leaf development. Overexpression of *BdGATA13* in *Arabidopsis* caused plants to be greener, due to higher chlorophyll content than in wild type controls (Figures 4, 6). It was shown previously that overexpression of *BdGATA4* (named *BdGATA15* in this study), *BdGATA6* (named *BdGATA18* in this study), and *SlGATA4*, *SlGATA5*, and *SlGATA7* from *S. lycopersicon* in *Arabidopsis* produced dark green leaves and accumulated high levels of chlorophyll when grown in light (Behringer et al., 2014). These results indicate that *BdGATA* genes have essential roles in chlorophyll biosynthesis.

In addition to regulating chlorophyll biosynthesis and chloroplast development *GATA* genes also function in seed germination, flowering time, and response to abiotic stress (Richter et al., 2010; Zhang et al., 2013). For example, the *Arabidopsis gnc* mutant flowered earlier than the wild type and overexpression of *GNC* showed a late-flowering phenotype (Richter et al., 2010); whereas in wheat, overexpression of *TaZIM-A1* caused delayed flowering under long-day conditions (Liu et al., 2019). Our results also showed that overexpression of *BdGATA13* caused delayed flowering in transgenic overexpression lines.

Real-time quantitative-PCR results showed that all *BdGAGA* genes were upregulated by PEG treatment, suggesting they function in response to drought. Overexpression of *BdGATA13* promoted drought tolerance in transgenic plants by regulating the expression of drought-related genes such as *RD29A*, *ABI5*, *LEA*, *NCED3*, and *DREB2A*. Consistent with *BdGATA13* results, overexpression of *OsGATA8* in rice increased tolerance to drought stress (Nutan et al., 2020), and overexpression of *SlGATA17* improved drought tolerance in tomato (Zhao et al., 2021).

Previous studies showed that *Arabidopsis GNC* is a transcriptional target downstream of GA (Richter et al., 2010). Some plant GATAs were induced by GA_3_. For example, the expression of *GmGATA58* was promoted by GA_3_ treatment (Zhang et al., 2020). In the present study, the expression levels of four GA-related genes in transgenic lines grown under normal conditions, namely *GA3ox1*, *GA2ox*, *GA20ox1*, and *GA20ox2*, were higher than in the WT. With GA_3_ treatment, expression levels of these genes in *BdGATA13* transgenic lines returned to normal levels, indicating that *BdGATA13* plays a role in plant growth by negatively regulating GA signaling. The overall results suggest that *BdGATA13* transcribes a TF that regulates various functions involved in abiotic stress and development in plants.

## Data Availability Statement

The datasets presented in this study can be found in online repositories. The names of the repository/repositories and accession number(s) can be found in the article/Supplementary Material.

## Author Contributions

JG analyzed the data and wrote the manuscript. XB, KD, and XY helped to carry out the experiments. PG and MZ contributed to writing the manuscript. CH and WS contributed to the experimental design, provided the advice for data analysis, and assisted in writing the manuscript. All authors have read and approved the final version.

## Conflict of Interest

The authors declare that the research was conducted in the absence of any commercial or financial relationships that could be construed as a potential conflict of interest.

## Publisher’s Note

All claims expressed in this article are solely those of the authors and do not necessarily represent those of their affiliated organizations, or those of the publisher, the editors and the reviewers. Any product that may be evaluated in this article, or claim that may be made by its manufacturer, is not guaranteed or endorsed by the publisher.

## Footnotes

1
http://web.expasy.org/protparam/

2
http://cello.life.nctu.edu.tw/

3
http://plants.ensembl.org/index.html

4
https://www.ncbi.nlm.nih.gov/

## Supplementary Material

Supplementary Figure 1 Maximum likelihood (ML) tree of GATAs in plants. The numbers of GATAs were 27 in *Brachypodium*, 29 in *Arabidopsis*, and 28 in rice, and construction was based on the full-length protein sequences. Four subgroups of GATAs were classified as I, II, III, and IV.
