# Supplementary figures and images for: Identification of GATA Transcription Factors in Brachypodium distachyon and Functional Characterization of BdGATA13 in Drought Tolerance and Response to Gibberellins
Source: Front Plant Sci. 2021 Oct 21;12:763665. doi: 10.3389/fpls.2021.763665 (PMC8567175; doi:10.3389/fpls.2021.763665)

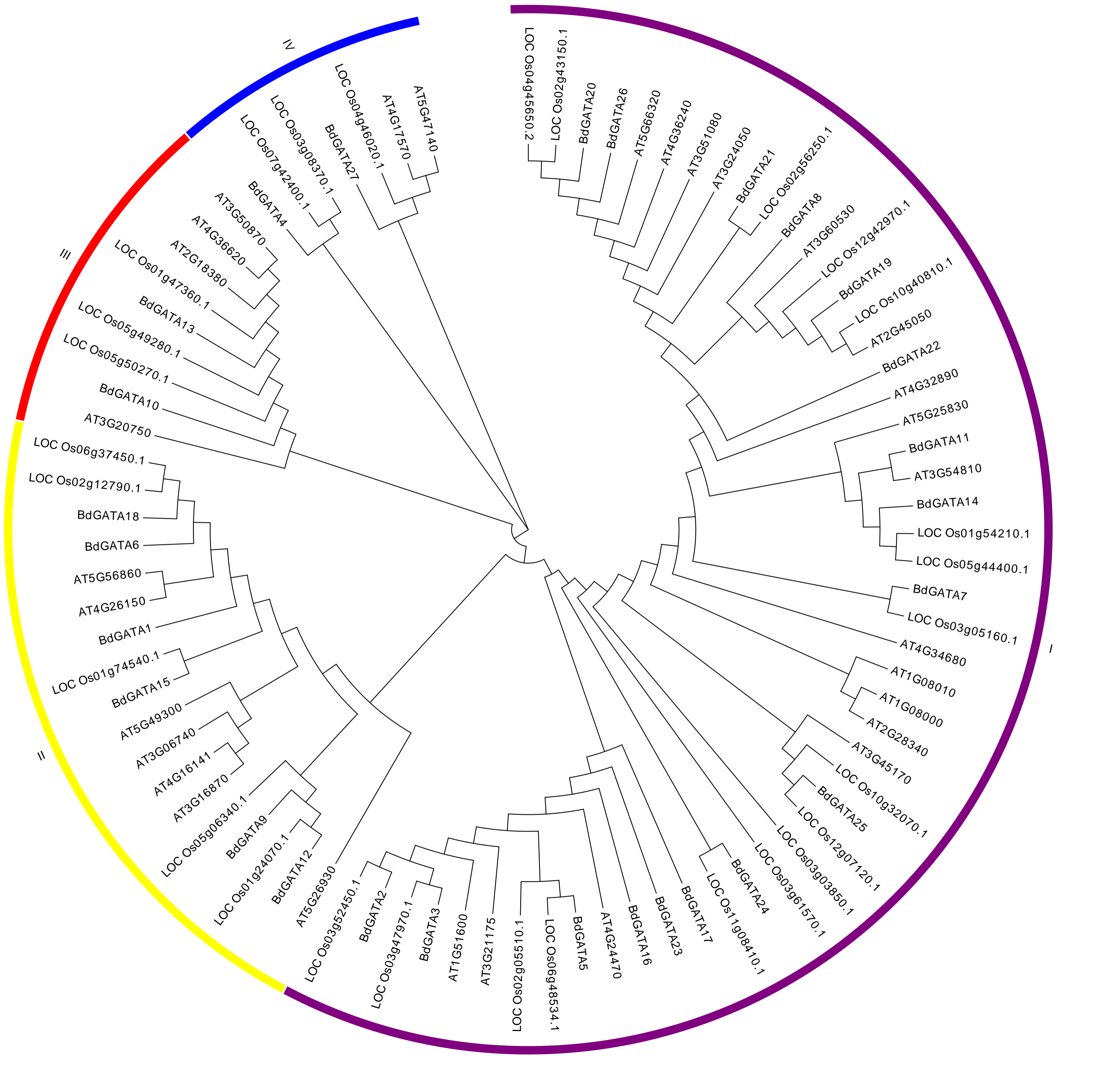

Supplement: Supplementary Figure 1 — Maximum likelihood (ML) tree of GATAs in plants. The numbers of GATAs were 27 in Brachypodium, 29 in Arabidopsis, and 28 in rice, and construction was based on the full-length protein sequences. Four subgroups of GATAs were classified as I, II, III, and IV. [file Image_1.pdf]
